# Bovine and degenerated human annulus fibrosus: a microstructural and micromechanical comparison

**DOI:** 10.1007/s10237-017-0900-z

**Published:** 2017-04-04

**Authors:** Claudio Vergari, Daniel Chan, Andrew Clarke, Jessica C. Mansfield, Judith R. Meakin, Peter C. Winlove

**Affiliations:** 1 School of Physics and Astronomy, University of Exeter, Physics Building, Stocker Road, Exeter, EX4 4QL UK; 2 Peninsula Spine Unit, Princess Elizabeth Orthopaedic Centre, Royal Devon and Exeter Hospital, Barrack Road, Exeter, Devon EX2 5DW UK

**Keywords:** Intervertebral disc, Fibres, Crimps, Second harmonic generation

## Abstract

The complex structure of the annulus fibrosus is strongly related to its mechanical properties. Recent work showed that it is possible to observe the relative movement of fibre bundles in loaded cow tail annulus; the aim of this work was to describe and quantify annulus fibrosus micromechanics in degenerated human disc, and compare it with cow tail annulus, an animal model often used in the literature. Second harmonic generation was used to image the collagen matrix in twenty strips of annulus fibrosus harvested from intervertebral disc of seven patients undergoing surgery. Samples were loaded to 6% tensile strain in 1% steps. Elastic modulus was calculated from loading curves, and micromechanical strains were calculated from the images using custom software. The same protocol was applied to twenty strips of annulus harvested from cow tail discs. Significant morphological differences were found between human and cow tail samples, the most striking being the lack of collagen fibre crimp in the former. Fibres were also observed bending and running from one lamella to the other, forming a strong flexible interface. Interdigitation of fibre bundles was also present at this interface. Quantitative results show complex patterns of inter-bundle and inter-lamellar behaviour, with inter-bundle sliding being the main strain mechanism. Elastic modulus was similar between species, and it was not affected by the degree of degeneration. This work gives an insight into the complex structure and mechanical function of the annulus fibrosus, which should be accounted for in disc numerical modelling.

**Electronic supplementary material:**

The online version of this article (doi:10.1007/s10237-017-0900-z) contains supplementary material, which is available to authorized users.

## Introduction

Annulus fibrosus is a strong fibrocartilaginous tissue that forms the outer ring of the intervertebral disc. It has a very complex structure: it is formed by series of concentric lamellae, discontinuous sheets of tightly packed bundles of collagen (Marchand and Ahmed 1990). These bundles are aligned within a lamella, but adjacent lamellae present different angles. The collagen fibres forming the bundles are mostly type I and II, and they appear crimped in the healthy disc (Eyre and Muir 1976; Gruber and Hanley 2002). The bundles are held together by a highly organized network of elastic fibres, while connectivity between lamellae is mainly maintained by localized trans-lamellar bridges (Schollum et al. 2008) and a distributed inter-lamellar matrix (Yu et al. 2007).

**Table 1:** Donors demographics and samples origin

Patient number	Gender	Age	Disc level	Samples harvested	Pfirrmann grade
1	Male	54	L5/S1	5	5
2	Female	66	L5/S1	2	5
3	Female	66	L5/S1	1	4
4	Female	36	L5/S1	3	5
5	Female	53	L5/S1	4	4
6	Female	40	L4/L5	3	5
7	Male	78	L5/S1	2	5

This architecture gives the annulus impressive mechanical properties: it can undergo fibre-associated strains up to 25% (Heuer et al. 2008) during physiological loading of the spine, without apparent damage. The mechanical behaviour of the annulus has been widely studied, both in its macroscopic and microscopic properties, from large strips of tissue (Acaroglu et al. 1995), down to the level of single lamella (Holzapfel et al. 2005; Monaco et al. 2016). It is also reported that the annulus fibrosus can undergo drastic structural and microstructural changes with degeneration. Its macroscopic tensile properties remain only marginally affected (Acaroglu et al. 1995), although its compressive properties can change, possibly as a result of tissue dehydration (Iatridis et al. 1998). However, a better understanding of annulus biomechanics is required to inform discussion of the aetiology and progression of disc pathology, particularly the processes of degeneration (Gregory et al. 2014).

Most previous work has inferred the mechanical behaviour of the tissue by tracking cells (Bruehlmann et al. 2004a, b) or markers that were glued or photobleached on the tissue (Bruehlmann et al. 2004a, b; Karakolis and Callaghan 2014; O’Connell et al. 2012), or alternatively relied on relatively low-resolution tissue tracking (for instance, 0.2 mm$^{2}$ elements (Baldit et al. 2014; Karakolis and Callaghan 2014)). Recently, advanced imaging techniques (second harmonic generation, SHG) were used to directly observe the kinematics of bundles and lamellae in loaded annulus (Vergari et al. 2016). SHG allows visualization of the collagen network without labelling or specific preparation of the sample; it has been applied to investigate, for instance, the structure and mechanics of cartilage (Cox et al. 2003; Mansfield et al. 2008), cornea (Thomasy 2014), tendon and ligament (Henninger et al. 2015; Legerlotz et al. 2014), as well as structural disorder in intervertebral disc (Reiser et al. 2007).

The aim of the present work was to investigate the microstructure and micromechanical properties of the degenerated human annulus fibrosus with SHG imaging, making comparisons with the bovine tail annulus as a model of a healthy tissue

**Fig. 1:**
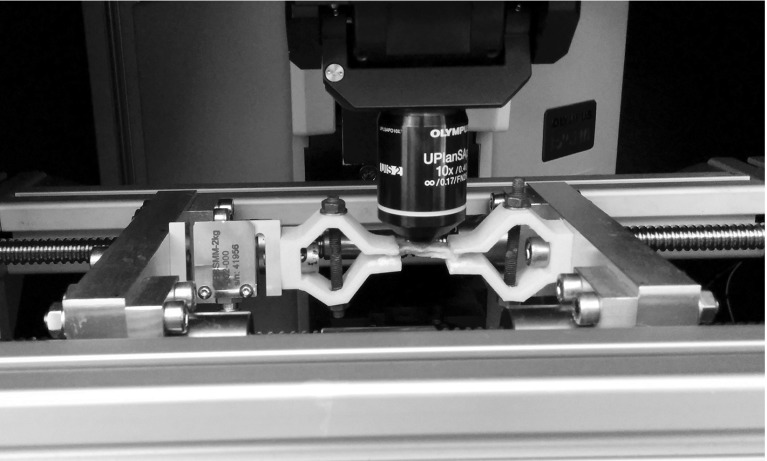
Mechanical testing rig for stretching of tissue samples under the microscope. A strip of annulus fibrosus is mounted between the grips

**Fig. 2:**
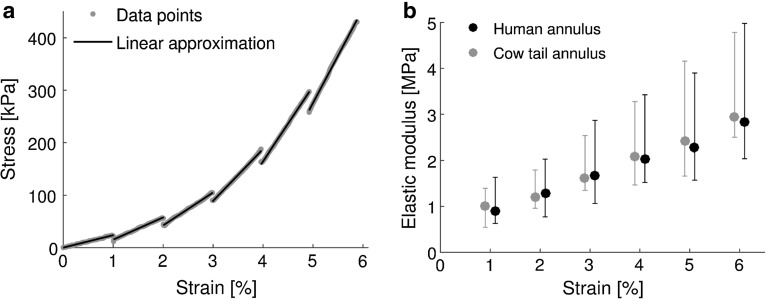
Typical stress vs strain curve of a strip of human annulus fibrosus (*left panel*); for each strain step, data were modelled with a piece-wise linear approximations, the slope of which represents the instantaneous elastic modulus. Imaging was performed between each step, and load relaxation occurred during this time (visible in the plot as a decrease in load between loading steps). Elastic moduli at each strain step are plotted for human and cow tail annulus in the *right panel* (median [1st and 3rd quartiles])

## Materials and methods

### Human samples

Seven patients undergoing spinal fusion were included in this study (56 ± 15 years old, Table 1). Patients were recruited to the ethically approved Royal Devon and Exeter Tissue Bank (REC ref: 11/SW/0018), which is set up to proactively collect and store “spare” tissue available from routine clinical procedures for forth-coming studies and is facilitated through the NIHR Exeter Clinical Research Facility.

The anterior portion of the L5/S1 ($n = 6$) or L4/L5 ($n = 1$, Table 1) disc was excised as part of the surgical routine to implant an anterior cage and immediately stored at −80${^{\circ }}$. A clinician analysed the patients’ magnetic resonance scans and classed the discs according to Pfirrmann grading (Pfirrmann et al. 2001).

Samples were thawed at room temperature on the day of the test and kept in phosphate-buffered saline solution (PBS). Strips of annulus were cut, with the length along the circumferential direction, through the thickness of the sample. Samples were measured by calliper once mounted in the testing rig. Average sample size was 11 mm length $\times$ 6 mm $\times$ 2 mm thickness, corresponding to 11 mm in circumferential direction, 6 mm in axial and 2 mm in radial direction. In some specimens, it was possible to cut sequential strips at increasing depths through the annulus so, in total, twenty strips of annulus from seven patients were tested (Table 1).

### Cow tail samples

Four fresh cow tails were obtained from the local abattoir and frozen intact at −25${^{\circ }}$; animals were about 2 years old. They were thawed at room temperature overnight before the test, intervertebral discs were excised, and twenty strips of annulus of the same dimensions as the human samples were prepared from eight discs.

### Tensile testing

Samples were kept in PBS between preparations and testing. A custom-built rig was utilized to load the samples (Fig. 1). The sample was glued between two 3D printed grips, one of which was attached to a 20 N load cell (DBBSMM, Applied Measurements Limited, Aldermaston, UK). The rig is motorized (stepper motor PKP264D14B-SG36-L, Oriental Motor, Tokyo, Japan) and can strain the sample in 17 $\upmu \hbox {m}$ steps. A preliminary calibration using test samples of known mechanical properties was performed to account for machine stiffness, allowing an accurate estimation of sample strain from crosshead displacement.

A preload of 0.2 N was applied to the sample, and force and strain were set to zero. Initial length ($L_0$), width (*W*) and thickness (*T*) of the sample were measured with callipers, and the sample was strained in 1% steps at 1 mm/min speed until 6% strain. Drops of PBS were applied to the sample immediately before the test to maintain hydration; the part of the test spent out of PBS bath lasted 6 minutes in average (11 max).

### Measurement of elastic modulus

Strain was calculated as $\varepsilon = ({L} - {L_{0}})/{L_{0}}$, where *L* is the instantaneous sample length measured during the tensile test. Stress was calculated as $\sigma = {F}$ / $({W}^*{T})$, where *F* is the load measured by the load cell and $W*T$ is the sample’s cross-sectional area. Stress vs strain curves are plotted (Fig. 2) and piece-wise linear approximations were fitted to each strain step; the slope of each line represents the instantaneous elastic modulus.

### Multiphoton imaging

**Fig. 3:**
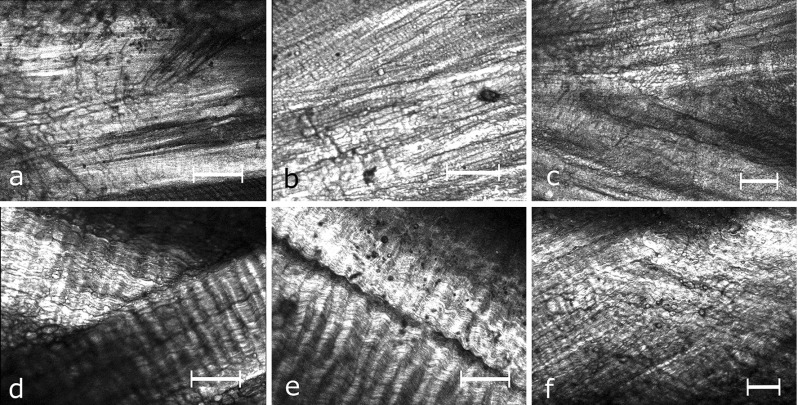
Typical examples of second harmonic generation imaging of human (**a**–**c**) and cow tail (**d**–**f**) annulus fibrosus (*white bar* $=$ 100 $\upmu$m). Images **b**–**e** show the interface of two lamellae. In **d**, fibres cross from one lamella to the other at the inter-lamellar interface

A previously described set-up (Vergari et al. 2016) was used for imaging the sample in the radial direction, with the sample orientated so that the *z*-axis aligned with the radius of the donor disc. The sample was illuminated with an 810-nm mode-locked femto-second Ti:Sapphire laser (Mira 900-D, Coherent Inc.) with a repetition rate of 76 MHz and a pulse width of 100 fs pumped by a 532 nm solid-state laser (Verdi V10, Coherent Inc.). Imaging of the backwards SHG excited in collagen fibres (Fig. 3) was performed using an Olympus Flouview BX51 microscope fitted with a 10$\times$/0.4NA air objective (Olympus UPlanS Apo).

Images were acquired at each step of the mechanical test (Fig. 3); the acquisition of an 800 $\times$ 600 pixels image (with sub-micron resolution corresponding to a rectangle of 0.4 $\times$ 0.3 mm to 0.8 $\times$ 0.6 mm size, depending on the zooming level) lasted about 20 seconds. Whenever possible, regions of interest (ROIs) showing the crossing of two lamellae were selected; such an ROI is richer in information than one showing a single homogeneous lamella because both the between-bundle and between-lamellae kinematics can be measured. Imaging position depended on where a surface flat enough to fit in the imaging plane over the whole ROI was observed; all ROIs were in the middle third of the sample.

### Image processing and strain calculation

Image processing was used to calculate the linear and shear strains within and between bundles of collagen and between lamellae, as previously described (Vergari et al. 2016). Briefly, custom software was written in MATLAB 2016b (The MathWorks Inc., Natick, MA) to obtain a displacement map from each series of images and calculate instantaneous microscopic strains. A region of interest (ROI) in the first image (sample at zero strain) was subdivided in square elements (between 12.8 and 28.8 $\upmu \hbox {m}^{2}$, corresponding to 167 elements in average), and each element was semi-automatically tracked in the subsequent images.

The horizontal ($${u}_{x})$$ and vertical displacements ($${u}_{y})$$ of the grid elements were smoothed with a local quadratic regression (span parameter $$=$$ 0.5) to reduce noise in the calculation of derivatives. Principal strains $$\varepsilon _{\mathrm{I}}$$ and $$\varepsilon _{\mathrm{II}}$$ were calculated by computing the eigenvalues ($$\varepsilon _{\min }$$ and $$\varepsilon _{\max })$$ of the strain tensor $$\bar{\bar{\varepsilon }}$$:

1 $$\begin{aligned} \bar{\bar{\varepsilon }} =\left[ {{\begin{array}{ll} {\frac{\partial u_x }{\partial x}}&{} {\frac{1}{2}\left( {\frac{\partial u_x }{\partial y}+\frac{\partial u_y }{\partial x}} \right) } \\ {\frac{1}{2}\left( {\frac{\partial u_x }{\partial y}+\frac{\partial u_y }{\partial x}} \right) }&{} {\frac{\partial u_y }{\partial y}} \\ \end{array} }} \right] \end{aligned}$$

Maximum shear ($\tau _{\mathrm{m}})$ was calculated at each grid point as: $\tau _\mathrm{m} =\frac{1}{2}\left( {\varepsilon _{\max } -\varepsilon _{\min } } \right)$ (Cruz Perez et al. 2015) and expressed as percentage value.

Finally, a segment was manually defined in each lamella or fibre bundle, aligned with the fibres. The ends of each segment were linked to the closest tracking elements, and their position was recalculated at each frame to follow the tissue deformation. This allowed the calculation of four parameters: the intra-bundle strain (i.e., the bundle’s lengthening or shortening), the linear strain between bundles (the bundles moving closer or away from each other), the local shear within the bundle (calculated from the displacement map) and the shear between bundles, quantifying their sliding. The latter was calculated as an angular strain and reported as percentage variation. Absolute strain values are reported.

### Statistics

Elastic modulus and microscopic strains were analysed by species and by degree of degeneration (multiple comparison considering cow tail as “no degeneration”). Strains were also analysed by type (intra-bundle, inter-bundle and inter-lamella). Effects of age on elastic modulus and strains were studied by correlation analysis. Correlations were quantified with Spearman’s rank correlation while differences between species were analysed with a Wilcoxon rank sum test to account for the non-normal distribution of data. Difference between multiple groups was analysed with Kruskal–Wallis tests (a nonparametric version of ANOVA) and Tukey’s post hoc analysis; significance was set at 0.05. Results are presented as median [1st quartile; 3rd quartile] to better represent the distribution of the data.

## Results

### Morphology

In cow tail samples, collagen fibres showed regular crimping and were organized within clearly delimited bundles (Fig. 3d–e). A ROI showing the crossing of two lamellae was found in eight out of twenty samples; in these, five samples showed sub-bundles of fibres interdigitating, with two lamellae fading into each other (Fig. 3f). The others showed clear delimitation of the two lamellae, although some fibres appeared to bend in running from one lamella to the other (Fig. 3d, Online Resource 1).

Five human discs were classed as Pfirrmann grade 5 (15 annulus samples) and two discs as Pfirrmann 4 (5 annulus samples). Human samples showed less SHG signal than cow tail, possibly indicating alteration of the collagen structure (Kim et al. 2000). In contrast to the bovine tissues, the collagen fibres were straight, with no crimping, and their arrangement appeared more disorganized (Fig. 3a, c). In particular, the bundles had less distinct boundaries, and clear inter-lamellar interfaces were only found in four samples out of twenty. Degenerated human samples showed no particular morphologic differences which could be related to the degree of degeneration as determined by Pfirrmann grading.

### Mechanical properties

Loading curves showed the characteristic nonlinear shape of collagenous biological tissues; the drops in load evident in Fig. 2a between each load step correspond to the imaging period, during which load relaxation occurred.

**Fig. 4:**
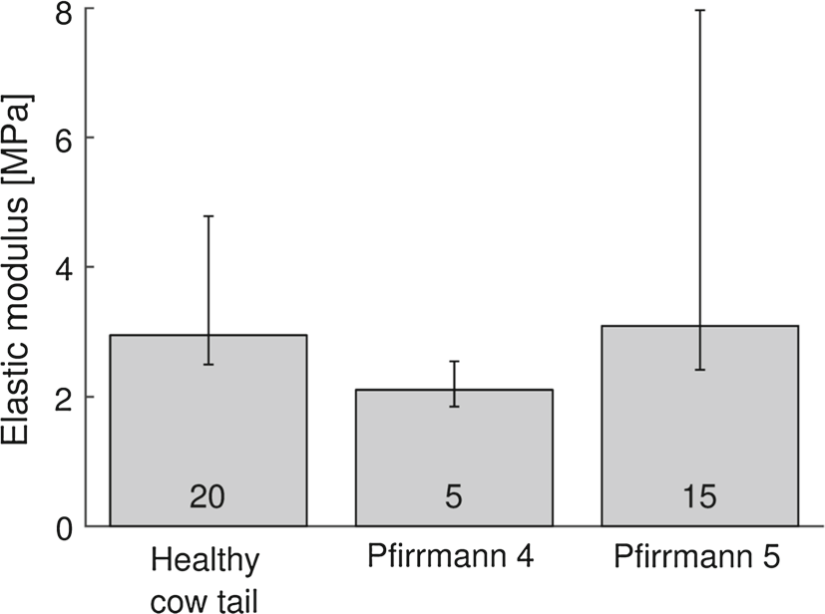
Relation of annulus elastic modulus at 6% strain with degeneration degree (zero corresponds to healthy cow tail disc) and patient age (*numbers* represent number of samples in each group). Neither the difference between degeneration groups was significant (Kruskal–Wallis test: $p = 0.1$) nor the correlation with age (Spearman’s rho $=$ 0.3, $p = 0.2$; *dashed line* is the linear regression)

Figure 2b shows the elastic modulus at each strain steps for cow tail and human annulus. The elastic modulus of the samples from the three degeneration groups (healthy cow tail, Pfirrmann grade 4 and 5) originated from the same distribution (Kruskal–Wallis test, $p = 0.1$); this implies that there was no significant effect of Pfirrmann grading on annulus elastic modulus nor any significant difference between human and cow tail samples. The difference between species remained non-significant when pooling Pfirrmann grades 4 and 5 (Wilcoxon rank sum test, $p = 0.88$). No effect of patient age was observed when pooling grades 4 and 5 either ($\rho = 0.3, p = 0.2$, Fig. 4).

### Microscopic behaviour

**Fig. 5:**
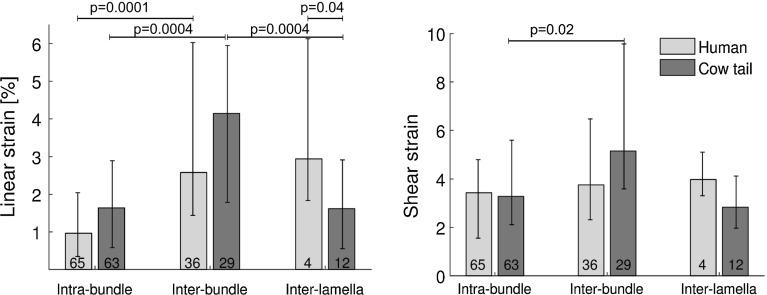
Microscopic linear and shear strain in human and cow tail annulus fibrosus. *Numbers* represent number of measurements; the sum is higher than the number of samples because several bundles and couples of bundles are visible in each sample. *p* values of significant differences are reported

All samples reached 6% applied strain with no apparent damage. At 6% applied strain, intra-bundle linear strain was significantly lower than inter-bundle strain both in human ($p = 0.0001$, Fig. 5) and cow tail sample ($p = 0.0004$); intra-bundle strain and inter-lamella strain were lower than inter-bundle strain in cow tail, but not in human ($p = 0.0004$). Inter-lamella strain was larger in human annulus (2.9% [1.8, 6.1]%) than in cow tail (1.6% [0.6, 2.9]%, $p = 0.04$), but for the human samples only four ROIs showing intersecting lamellae were found. In cow tail samples, intra-bundle shear (3.3% [2.1, 5.6]%) was significantly lower than inter-bundle shear (5.2% [3.6, 9.6]%, $p = 0.016$). All other unmarked comparisons in Fig. 5 did not show significant differences.

**Fig. 6:**
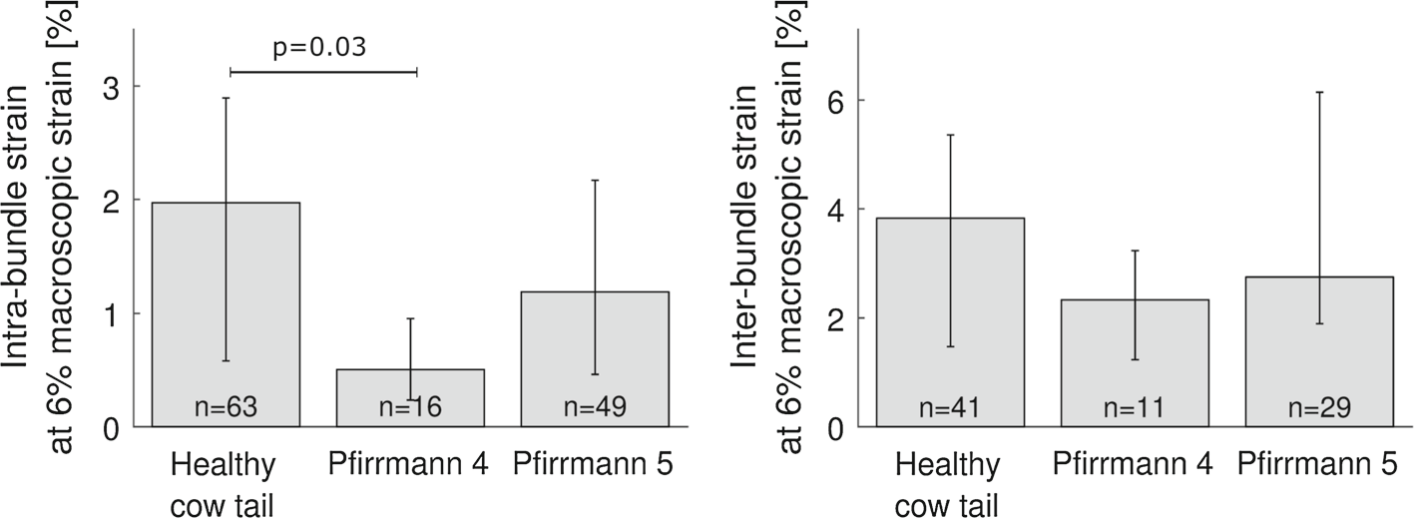
Intra- and inter-bundle linear strain according to degree of degeneration. Intra-bundle strain was significantly higher in healthy annulus (cow tail) than in human Pfirrmann grade 4 annulus ($p = 0.03$). *Numbers* represent number of measurements; the sum is higher than the number of samples because several bundles and couples of bundles are visible in each sample

Intra-bundle strain in cow tail was slightly higher than in the five human samples that were graded as Pfirrmann 4 ($p = 0.03$, Fig. 6). Degree of degeneration did not show other significant effects ($p > 0.05$, Fig. 6); inter-lamella strain was not tested because of an insufficient number of sample in each group.

**Fig. 7:**
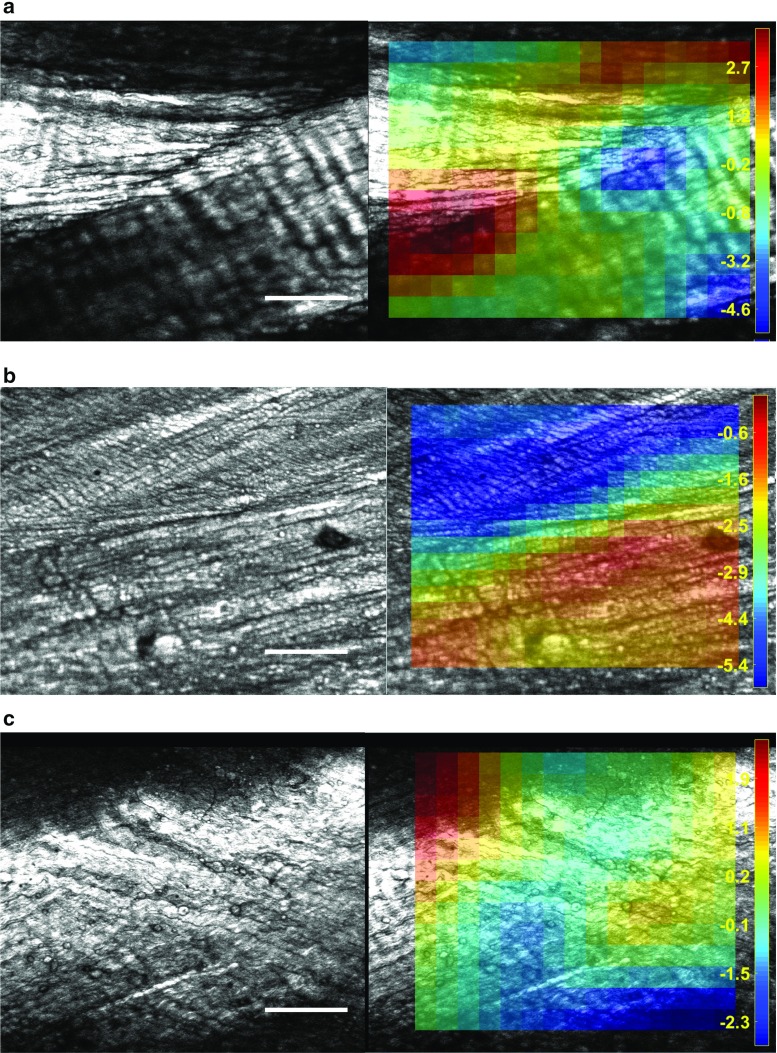
Maximal shear in three examples of inter-lamellar crossing at 6% applied strain in the horizontal direction (*white bar* $=$ 100 $\upmu \hbox {m}$). **a** ($13 \times 17 = 221$ tracked elements) shows a cow tail sample with a sharp separation between lamellae and strain concentration at the junction. **b** ($16 \times 20 = 320$ tracked elements) shows a human sample, with homogenous strain in each lamella. **c** ($13 \times 15 = 195$ tracked elements) shows an example of cow tail sample with interdigitation of fibre sub-bundles and a transition of shear strain between lamellae. Animations of the strain steps are available for each panel as Online Resources 1–3

Figure 7 shows the distribution of maximum shear strain in three examples of inter-lamellar crossing. Maximum shear strain ranged between −49 and 3.8% in panel a, −4.3 and 0.6% in panel b and −2.8 and 2.6% in panel c. Online Resource 1 (Fig. 7a) shows the intersection of two large bundles of fibres running at a relative angle of 133${^{\circ }}$ in a cow tail sample. Fibres link the two lamellae, bending and running from one to the other. In this transition region, crimp straightens with loading, whereas the crimp within the bundle remains unchanged. Maximum shear is concentrated along this transitional zone, while it is much lower within the two bundles. No slipping occurs between lamellae.

Online Resource 2 (Fig. 7b) shows two lamellae intersecting with an angle of 10${^{\circ }}$ in a human sample. Each lamella shows a homogeneous maximum shear, with no shear concentration at the interface. Both lamellae realign towards the direction of the applied strain.

Online Resource 3 (Fig. 7c) shows the interdigitation of two lamellae in a bovine sample, intersecting at about 45${^{\circ }}$. Maximum shear smoothly transitions between the two lamellae. Bundles in this lamella are lengthening, with a modest uncrimping of the fibres; this uncrimping accompanies the realignment of the second lamella, and no sliding occurs between lamellae. Again, the sub-bundles of the lamella at the top right bend at their ends, towards the orientation of the other lamella.

## Discussion

**Fig. 8:**
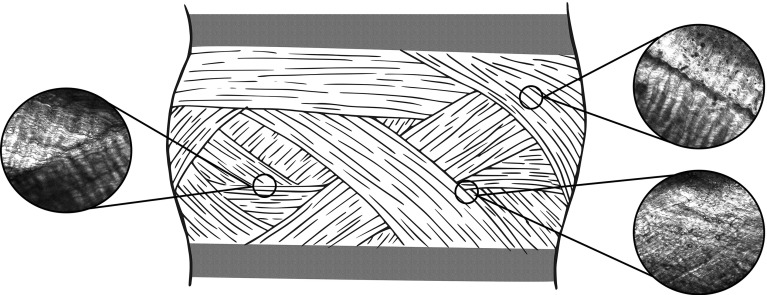
Schematic drawing of intervertebral disc structure. Lamellae are incomplete, formed by bundles of crimped fibres running in several different directions. Lamellae can cross over each other or fuse with interdigitation of bundles

Inter-lamellar behaviour has usually been imaged in the circumferential or axial direction, thus visualizing the lamellar cross sections. In the present work, samples were imaged in the radial direction. Visualization of inter-lamellar interface was possible because lamellae are discontinuous, i.e., they do not run in a single layer all around the disc (Marchand and Ahmed 1990) (Fig. 8). If, *ad absurdum*, layers were continuous, (1) no inter–lamellar interface would exist in the middle of the annulus and (2) it would not be possible to image two lamellae simultaneously in the radial direction (as the more external lamella would hide the adjacent inner lamella).

Two types of interfaces were found: a distinct separation between lamellae (Fig. 3b–d) or an interdigitation of sub-bundles (Fig. 3f). These interfaces must be very thin and correspond to a thinning of the lamella at its end, because the depth of field of the SHG set-up was 7.2 $\upmu \hbox {m}$ (as calculated from the equation given by Squier and Müller 2001). This is quite thin compared to the lamellar thickness, which is 280 ± 90 $\upmu \hbox {m}$ in adult humans and 157 ± 83 $\upmu \hbox {m}$ in young cow tail (Adam et al. 2015). The ends of discontinuous lamellae, where these inter-lamellar boundaries occur, indeed tend to thin out (Adam et al. 2015), which explains why imaging these boundaries was possible. Older human lamellae are thicker than cow tail lamellae, which might be the reason why inter-lamellar interfaces were rarer in human samples: if the interface is too thick, it cannot be imaged in a single two-dimensional image.

Fibres bending and directly linking lamellae (Fig. 3d, Online Resource 1) have not been previously reported, but the existence of such stiff structures can explain why no sliding occurs at the inter-lamellar interface. Similar structure and mechanics can be seen in the Online Resource 1 of a previous work, which employed a different experimental protocol (Vergari et al. 2016). In that work, large sliding occurred between bundles of fibres in cow tail samples. In the present work as well, inter-bundle shearing (5.2% [3.6, 9.6]% median [1st, 3rd quartiles]) was significantly higher than intra-bundle shearing (3.3% [2.1, 5.6]%, $p = 0.02$) confirming that inter-bundle sliding is the main straining mechanisms. Bundles in human samples did not appear to slide, as confirmed quantitatively by the values of intra- and inter-bundle shearing (3.4 and 3.8%, respectively), which showed that the same shearing occurred within the bundle as between bundles.

Healthy young cow annulus and adult human annulus showed different microstructure. The most striking difference is the complete lack of fibre crimping in human annulus. Fibre crimping in the annulus has been described for cow tail, canine and human annulus (Bruehlmann et al. 2002; Cassidy et al. 1989, 1990); the study by Cassidy et al. on human annulus, however, does not report whether the included discs were healthy. Therefore, it is possible that the lack of crimps is an effect of disc degeneration. Tendons show crimped structure of collagen fibres, aligned with its main axis. Crimp angle at rest can decrease with age and exercise in equine superficial digital flexor tendon (Patterson-Kane et al. 1997a, b). However, only a small reduction in angle was reported, not a complete loss of crimping, and in human Achilles tendon no effect of rupture was observed on crimp angle, apart from a straightening at the site of the rupture. (Magnusson et al. 2002; Järvinen et al. 2004). On the other hand, loss of crimp and a small reduction in crimp angle was described in ruptured canine ligament (Hayashi et al. 2003). Still, the comparison of annulus fibrosus and tendon crimps is not appropriate since crimps in tendon have a clear mechanical role: they increase the structure’s initial compliance by straightening.

Crimp in healthy cow tail can straighten if a single lamella is loaded in the direction of the fibres (Pezowicz et al. 2005). This straightening within bundles of fibres, however, did not occur at 6% applied strain of larger multi-lamellar samples tested in the present work, nor did it occur in a previous study with strains up to 28% (Vergari et al. 2016). Therefore, it is unlikely that fibres can straighten *in vivo* under physiological loading, although it must be noted that both these works used tail discs; cow lumbar discs might have a different behaviour. This might mean that the lack of crimp in degenerated human disc does not necessarily imply a lack of initial compliance for the lamella. In fact, straight parallel fibres can slide more easily against each other, thus increasing the lamellar compliance. While this hypothesis is still speculative, it is corroborated by the same shearing observed within the bundle as between bundles in degenerate human samples.

Crimp, however, appeared to be straightening at the interface between two lamellae (Online Resource 1), facilitating the large reorientation of the lamellae. It is possible that fibre crimp act locally as a torsional spring, making the inter-lamellar interface strong, allowing no sliding, but at the same time being very flexible.

The schematic drawings of intervertebral disc structure usually reported in the literature, depicting the annulus as a neat sequence of lamellae that run around the whole disc, with precise alignment of fibres at ±30${^{\circ }}$, are not consistent with our newer observations. In Fig. 8, we propose an update on the drawing by Burke (1969) depicting incomplete lamellae running in several different direction, with inter-lamellar intersection and regions of possible interdigitation of bundles. These aspects should be included in biomechanical numerical model of the disc, as they likely have a significant impact on inter-lamellar mechanics (Nerurkar et al. 2011). Further research should elucidate on how these inter-lamellar structures change along the radial direction.

All human samples were harvested from degenerated discs. Acaroglu et al. (1995) observed a slight decrease in elastic modulus with degeneration, albeit not significant, and this is consistent with the present results showing that degeneration had very small effects. However, all discs were highly degenerated (Pfirrmann grade 4 or 5). It is possible that mechanical differences between these two grades of severe degradation are small and that including a wider range of Pfirrmann grades would have highlighted differences with the less severely degenerated discs. Also, compressive properties of the annulus might be more sensitive to degeneration than tensile properties (Iatridis et al. 1998).

A limitation of the present work is that testing and imaging were not performed in a saline bath. Preliminary tests showed that annulus sample swell significantly in saline, this reduces the flatness of the sample’s surface, making imaging impossible because of the thin imaging depth of field. The protocol adopted in which drops of PBS were applied to the samples once they were mounted in the testing device was felt to provide the best compromise between swelling and dehydration. A preliminary test was performed by mounting a cow tail annulus sample on the rig, loading it at 1% strain and letting it rest for two hours (much longer than the average 6 minutes of the “dry” testing phase of this work). No microscopic strain change was observed during this time. This test also verified that dehydration did not affect the microstructural features described in this work.

Another limitation is that cow tail discs were adopted as healthy control for degenerated human discs. Healthy human discs are usually not excised so no such surgical residual was available. On the other hand, degenerated discs are not usually found in young cow tail. Nevertheless, cows are often used in the literature as animal model replacement for human disc biomechanics.

Applied strains did not exceed 6%, so the mechanical properties reported are relative to the toe region of the stress strain curves. The median elastic modulus at 6% applied strain was 2.7 MPa in cow tail annulus and 2.8 MPa in human samples; mean and standard deviation values were 5.0 ± 5.0 and 2.9 ± 1.7 MPa, respectively. Previous work has reported similar mean values for elastic modulus: O’Connell et al. estimated a modulus of 7.33 ± 5.50 MPa for human lumbar disc annulus strips (2.1 ± 0.3 mm thickness) (O’Connell et al. 2012). Elliott and Setton (2001) reported 2.52 ± 2.27 MPa on thicker samples (5 mm) of human lumbar annulus. This high inter-sample variability is likely due to a combination of factors: differences between subjects (age, medical history, pathology, etc.), spinal level, but also samples size measurement error, difficulties in mechanical testing reproducibility, etc.

Single lamella testing of bovine tail annulus yielded a much lower elastic modulus of 1.88 ± 0.6 MPa (mean ± standard error, Monaco et al. 2016); these samples, however, were loaded in a direction orthogonal to the fibres, thus loading the weaker inter-bundle network. Consistent with the orthotropic nature of the tissue, single-lamella elastic modulus along the direction of the fibres is much higher (53.2 ± 27.5 MPa (Pezowicz 2010)).

## Conclusion

Annulus micromechanical behaviour has been described and quantified with unprecedented detail, and a number of additional features were observed: interdigitation of fibre bundles at inter-lamellar interfaces, and fibres bending to run from one lamellae to the other. A new function has also been hypothesized for fibre crimp: it could act as a spring at the inter-lamellar interface to facilitate the realignment of the lamellae, supporting the role of elastic inter-lamellar connections. This structure could to have a major impact on disc biomechanics and should be accounted for in numerical modelling.

## Electronic supplementary material

Below is the link to the electronic supplementary material.

Supplementary material 1 (mp4 1239 KB)

Supplementary material 2 (mp4 1696 KB)

Supplementary material 3 (mp4 1627 KB)
